# M1 of Murine Gamma-Herpesvirus 68 Induces Endoplasmic Reticulum Chaperone Production

**DOI:** 10.1038/srep17228

**Published:** 2015-11-30

**Authors:** Jiaying Feng, Danyang Gong, Xudong Fu, Ting-ting Wu, Jane Wang, Jennifer Chang, Jingting Zhou, Gang Lu, Yibin Wang, Ren Sun

**Affiliations:** 1 Department of Molecular and Medical Pharmacology, University of California, Los Angeles, California 90095.; 2 Zhejiang University, Hangzhou, People’s Republic of China; 3 Department of Anesthesiology, University of California, Los Angeles, California 90095.

## Abstract

Viruses rely on host chaperone network to support their infection. In particular, the endoplasmic reticulum (ER) resident chaperones play key roles in synthesizing and processing viral proteins. Influx of a large amount of foreign proteins exhausts the folding capacity in ER and triggers the unfolded protein response (UPR). A fully-executed UPR comprises signaling pathways that induce ER folding chaperones, increase protein degradation, block new protein synthesis and may eventually activate apoptosis, presenting both opportunities and threats to the virus. Here, we define a role of the MHV-68M1 gene in differential modulation of UPR pathways to enhance ER chaperone production. Ectopic expression of M1 markedly induces ER chaperone genes and expansion of ER. The M1 protein accumulates in ER during infection and this localization is indispensable for its function, suggesting M1 acts from the ER. We found that M1 protein selectively induces the chaperon-producing pathways (IRE1, ATF6) while, interestingly, sparing the translation-blocking arm (PERK). We identified, for the first time, a viral factor capable of selectively intervening the initiation of ER stress signaling to induce chaperon production. This finding provides a unique opportunity of using viral protein as a tool to define the activation mechanisms of individual UPR pathways.

Molecular chaperones are a group of proteins that possess the ability to transiently assist in the folding and assembly of other macromolecules. They play essential roles in maintaining cellular homeostasis through multiples biological processes such as dissembling polypeptide aggregates, transporting proteins across membranes and escorting proteins for degradation^1^,^2^. Most intracellular chaperones function as housekeeping proteins and are constitutively expressed in non-stressful situations. Nevertheless, in response to environmental fluctuation, the chaperones can be drastically upregulated to provide cytoprotection against the stress conditions including virus infection^3^. This is particularly true for the chaperones residing in the lumen of endoplasmic reticulum^2^.

The endoplasmic reticulum (ER) plays a central role in protein synthesis, folding, assembly with the help of a large set of ER-resident chaperones^2^. Multiple disturbances that alter ER homeostasis, such as calcium dysregulation, glucose deprivation and viral infection, can cause accumulation of misfolded/unfolded proteins that exceeds the folding capacity of the ER and elicits the evolutionarily conserved unfolded protein response (UPR)^4^,^5^,^6^,^7^. Through a collection of ER-to-nucleus signaling pathways that control specific gene expression, the UPR is designed to re-establish homeostasis in the ER lumen. Notably, if UPR prolongs and cells are unrecovered, apoptosis will be triggered. Up to date, three distinct UPR signaling pathways have been identified, with each arm individually mediated by three ER membrane-bound stress sensors: inositol-requiring protein-I (IRE1), activating transcription factor-6 (ATF6) and protein kinase RNA (PKR)-like ER kinase (PERK). It remains controversial on how the three signaling proteins sense the ER stress^8^. One prevailing theory is that they are bound by ER resident chaperones in un-stressed conditions, and become activated when the excess unfolded proteins compete away the associated chaperones^7^. However, recent work indicated that the each transmembrane signal transducer may possess unique properties in sensing the stress, and the state of chaperone association is not sufficient to determine their activation statues^9^. These intriguing findings have raised important questions that the field urges to have an answer.

As UPR initiates, the IRE1 oligomerizes and autophosphorylates the juxtaposed kinase domain. It subsequently activates its endoribonuclease function to remove a 26-nt intron from the precursor X-box-binding protein 1 (XBP1) mRNA. The spliced XBP-1 mRNA encodes a potent transcription factor that further activates UPR genes (e.g.: ERdj4) in the nucleus^6^. PERK activation resembles IRE1 as it undergoes oligomerization upon stress, induces autophosphorylation and activates its kinase domain. Active PERK phosphorylates and inactivates the eukaryotic translation factor-2 (eIF2α), attenuating global protein synthesis and thereby reducing the amount of new polypeptides entering the ER^6^. Unlike IRE1 and PERK, ATF6 is first transported from the ER to the Golgi under stress, where its cytosolic domain is released by protease cleavage and moves to the nucleus^10^. The nuclear ATF6 acts as a transcription activator of XBP1, and ER chaperone genes GRP78 and GRP94^11^,^12^. It is important to note that the production of various ER chaperones is coordinated by the crosstalk between the three signaling branches. Previous studies have shown that under ER stress, GRP78 and GRP94 production is principally induced by the ATF6 pathway but is also partially controlled via the IRE1 pathways; likewise, although the IRE1 branch has a dominant impact on the induction of the ERdj4 gene, the ATF6 pathway is believed to play a role as well; moreover, XBP1, the central player of the IRE1 axis, is produced downstream of the ATF6 pathway^13^,^14^,^15^.

Viruses are intracellular parasites. They depend on host apparatuses and cellular processes to support productive infection. Also, viruses are evolved to cope with the rapidly changing environment in the host. During the course of infection, a large amount of viral proteins are synthesized in a short period. Such demand pushes the cellular folding capacity to its upper limit which in turn can become a restricting factor to viral propagation. Therefore, most successful pathogens have evolved mechanisms to interact with the host chaperone network in order to create a favorable environment. For instance, the simian virus 40 utilizes chaperones for uncoating and entry into the host cells^16^; the hepatitis C virus (HCV) requires the heat shock protein 90 (Hsp90) chaperone for protease maturation^17^; both HCV and HBV are reported to involve a number of ER chaperones for viral protein folding^18^,^19^; and the human immunodeficiency virus type 1 (HIV-1) relies on chaperone cyclophilin A for virion assembly^20^.

The herpesviridae is a family of large DNA viruses known for their ability to establish lifelong infection in natural hosts. The family is further divided into the alpha-, beta- and gamma- subgroups of herpesviruses. The gamma-herpesviruses can establish latent and lytic infection in the host. Two known human gamma-herpesviruses are Epstein-Barr virus (EBV) and Kaposi’s Sarcoma-associated Herpesvirus (KSHV). Persistent infections of gamma-herpesviruses are associated with a variety of human diseases including Burkitt’s lymphoma, Hodgkin’s disease, nasopharyngeal carcinoma, and Kaposi’s sarcoma^21^. The narrow host range of human gamma-herpesviruses has limited their pathogenetic studies *in vivo*. We and others have been using murine gamma-herpesvirus 68 (MHV-68) as a model virus to study gamma-herpesvirus infection^22^. MHV-68 bears considerable genetic and biological resemblance to EBV and KSHV. However, unlike human gamma-herpesvirus, MHV-68 is capable of establishing robust infection *in vitro* and *in vivo*^23^.

M1 is a unique gene exclusively encoded by MHV-68. M1 exhibits a 25% identity and 45% similarity to M3, another MHV-68 specific open reading frame (ORF). Previous studies have demonstrated that both M1 and M3 are nonessential for MHV-68 infection *in vitro* and *in vivo*^24^,^25^,^26^. Only a few studies have reported on the extracellular function of the M1 protein^24^,^27^,^28^ whereas its intracellular activity remains unknown. In this study, we defined a role of M1 in controlling ER chaperone production through analysis with protein ectopic expression and usage of a recombinant MHV-68. Our findings on M1 gene in interacting with the host chaperone system provide new implications in how virus can selectively deregulate cellular pathways amongst the complex viral-host interaction.

## Results

### M1 induces the expression of ER chaperone genes

**Figure 1:**
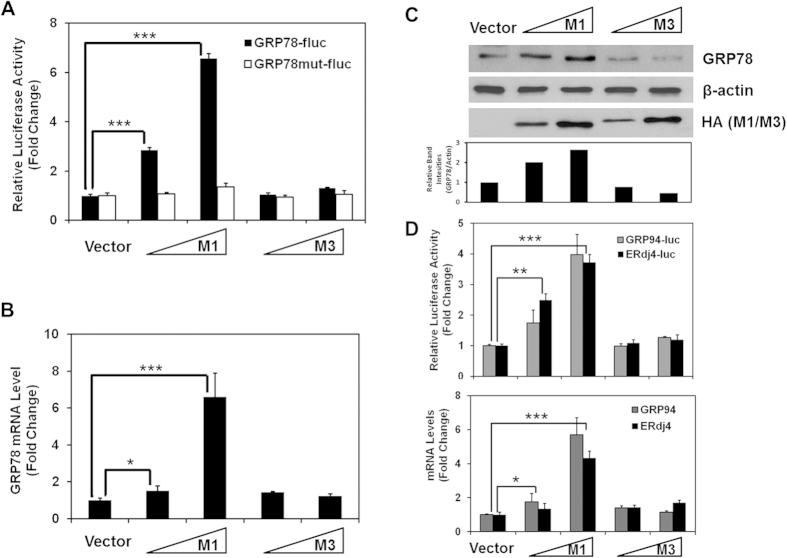
M1 induces expression of ER chaperone genes. (**A**) 293T cells were transfected with the GRP78-fluc or GRP78mut-fluc reporter plamids, the PGK-renilla-luciferase as an internal control, and increasing amounts of plasmids encoding M1 or M3. The cell lysates were prepared 24 hours posttransfection for dual-luciferase assay. The ratio of firefly luciferase activity to renilla luciferase activity was calculated based on the value of the vector control (set as 1). This assay and all following assays were performed in triplicates. Error bars show standard deviation. **(B)** Cells were transiently transfected with the M1 or M3 expression plasmids for 24 hours and were harvested for RNA extraction. Quantitative RT-PCR was performed using a primer set specific for GRP78 (Table 1). The levels of GRP78 mRNA were normalized to that of GAPDH mRNA. The fold changes shown were calculated relative to the values obtained in vector-transfected cells (set as 1). **(C)** Cells were transfected as described in Fig. 1B and were harvested for western blot analysis using antibodies specific for GRP78 and β-actin (loading control) as indicated. M1 and M3 genes were tagged with Human influenza hemagglutinin (HA) and the expressions were shown using anti-HA antibodies. The lower panel shows the intensity of bands that was quantified from western blot analysis by measuring the peak height of the bands using ImageJ software. The intensity of bands in top and middle panel was compared to the intensity of vector control (which was defined as 100%). The relative intensity was calculated by normalizing the intensity of upper panel bands (GRP78) to the middle panel bands (β-actin). **(D)** The promoter activities of GRP94 and ERdj4 were determined by reporter assay as described in Fig. 1A using the GRP78-fluc and ERdj4-fluc reporter plasmids, and mRNA levels determined quantitative RT-PCR analysis as in Fig. 1B using specific primer sets for indicated genes (Table 1). *P < *0.05*; **P < *0.01*; ***P < *0.001 by Student’s t test.*

In order to systematically identify viral components of MHV-68 that modulate the expression of ER chaperone genes, we conducted a genomic viral ORF screen using a reporter system based on GRP78 expression. GRP78 is the most abundant ER resident chaperone and is highly stress-inducible^29^. The reporter construct (GRP78-fluc) used in the study contains the promoter region of GRP78 driving the coding sequence of firefly luciferase gene^30^,^31^. GRP78-fluc was co-transfected into the 293T cells with PGK_renilla-luciferase (an internal control plasmid in which the renilla luciferase expression is driven by the constitutively active PGK promoter), and either individual ORFs of MHV-68 or a vector control. The reporter activity was measured by dual-luciferase assay 24 hours post transfection. From the screen, a strong induction (>6-fold) on the reporter was consistently noted with multiple clones of M1 (Fig. 1A and data not shown); in contrast, transfection with the sequence-related M3 showed basal-level activity comparable to that of vector control. The observed changes in reporter activity were specific for the stress response elements within the promoter region of GRP78, because M1 had no effect on the mutant reporter plasmid (GRP78mut-fluc) in which the response elements were eliminated^30^. In line with the reporter assays, the endogenous mRNA and protein levels of GRP78 were also upregulated in a dose-dependent manner with M1 expression as shown, respectively, by real time RT-PCR (Fig. 1B) and western blot analyses (Fig. 1C).

Since M1 protein expresses to a high level in transfected 293T cells, it may be argued that the induction of the chaperone was simply due to protein overload in the ER. However, the equally high expression of M3 protein (Fig. 1C) did not elicit the same response rendering this possibility unlikely. It should also be noted that when performing a titrated transfection of the plasmid encoding M1, strong activation of the chaperones genes was observed even with a small amount of the plasmid (0.06 ug/60 mm-dish) however no effect was seen in cells transfected with maximal amount of the M3-encoding plasmid (2.4 ug/60 mm-dish) (Fig. 1A and data not shown).

**Figure 2:**
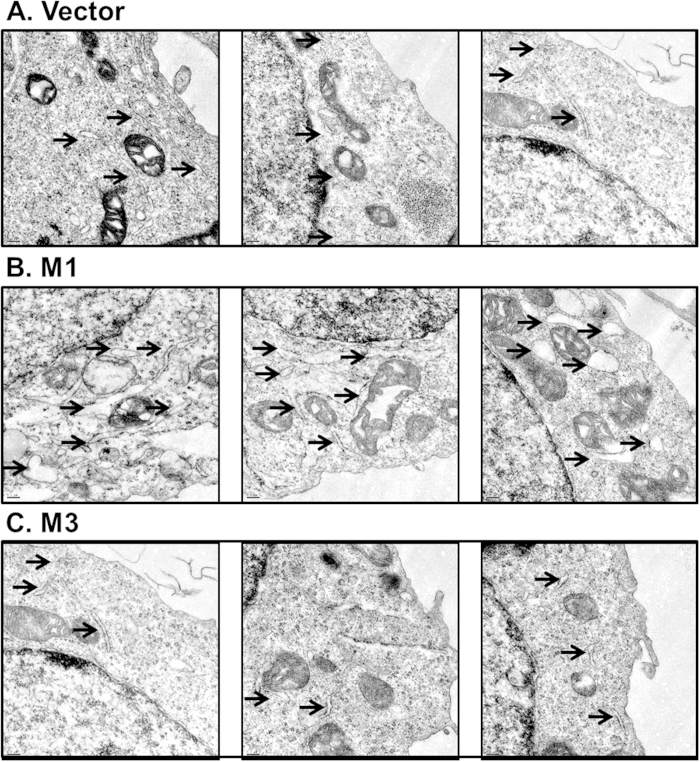
M1 induces ER expansion. 293T cells were transfected with vector control **(A)** or plasmids encoding M1 **(B)** or M3 **(C)** for 24 hours, and were prepared for electron microscopy analysis. Arrows point to representative ER.

In addition to increased production of GRP78, we also observed significantly enhanced gene expression of the other two major UPR chaperones GRP94 and ERdj4 in the presence of M1 (Fig. 1D). These findings directed our attention to the changes in the ER morphology. We speculated that in order to accommodate the newly-synthesized folding machineries, the amount or the size of the ER must have changed dramatically as described previously^32^. Indeed, electron microscopy analysis revealed a massive expansion of the ER in the M1-expressing cells as compared to cells transfected with the M3 plasmid or the control vector (Fig. 2).

### ER localization of M1 is indispensible for ER chaperone induction

In order to gain insights into the mechanisms by which M1 induces ER chaperone expression, we examined the cellular localization of M1 protein. Sequence analysis of M1 and M3 showed that both proteins contain signal peptides and cleavage sites located close to the N-terminus. The results also indicated that both M1 and M3 are secreted proteins, an observation confirmed in our study (Fig. S1) and by other groups^25^,^28^. It has been well established that in eukaryotic cells, nascent proteins destined for secretion first enter the ER, where they are modified and assembled prior to transit into the secretory pathway. Indeed, both M1 and M3 proteins localize to the ER, as evidenced by significant co-distribution of the HA (M1/M3) and concanavalin A (Con A) signals (see below). Conc A is a probe reported to recognize the residues of α-mannopyranosyl and α-glucopyranosyl commonly found in the ER and Golgi apparatus.

**Figure 3:**
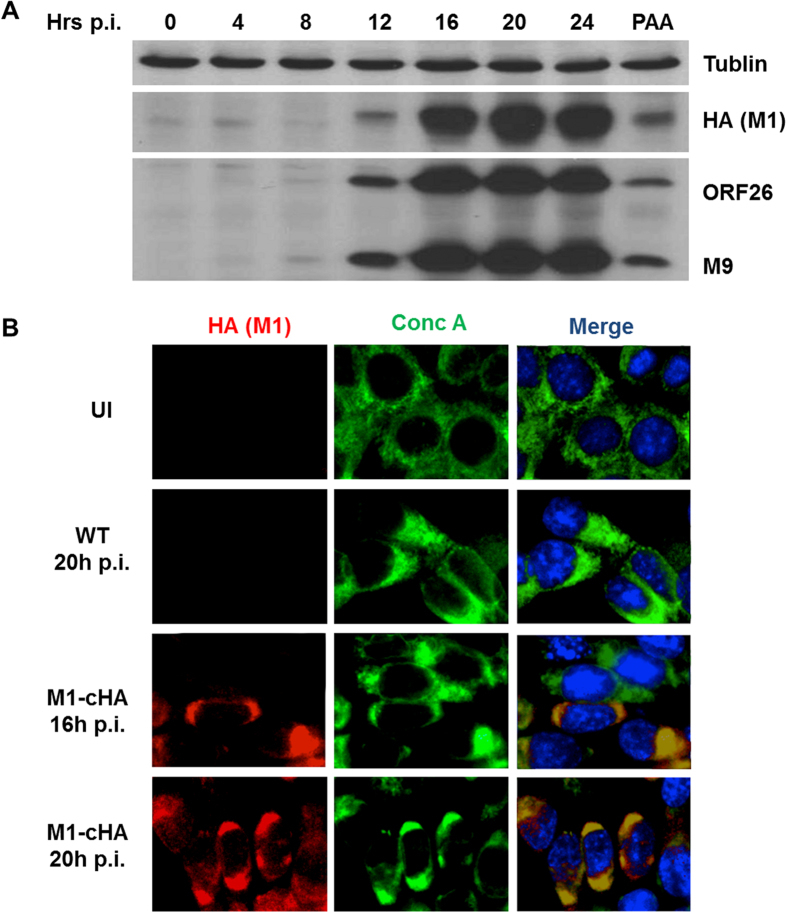
M1 localizes to cellular ER during MHV-68 infection. (**A**) NIH3T3 cells were infected with M1-cHA MHV-68 at MOI 10 and harvested at indicated time points for western blot analysis using antibodies specific for tublin (top), HA (middle), ORF26 and M9 (bottom). (**B**) NIH3T3 cells were mock-infected or infected with wild-type (WT) or M1-cHA MHV-68 at MOI 10 and fixed for IFA analysis as described in Materials and Methods using antibodies against HA (red), concanavaline A (conc A) (green) and Hoechst (Blue).

To determine whether in the context of viral infection that M1 protein also enters ER, we constructed a recombinant MHV-68 (M1cHA) with an HA tag added to the C-terminus of the M1 ORF. The tagged virus replicates normally (Fig. S2) and enables detection of the M1 protein during infection. M1 expression was induced at 12 hours post infection in the NIH3T3 cells, in accordance with the production of viral capsid proteins ORF26 and M9 (Fig. 3A). In addition, we observed significant a co-localization of M1 with Conc A at its peak expression in the infected NIH3T3 cells (Fig. 3B), suggesting the presence of M1 protein in the ER lumen.

**Figure 4:**
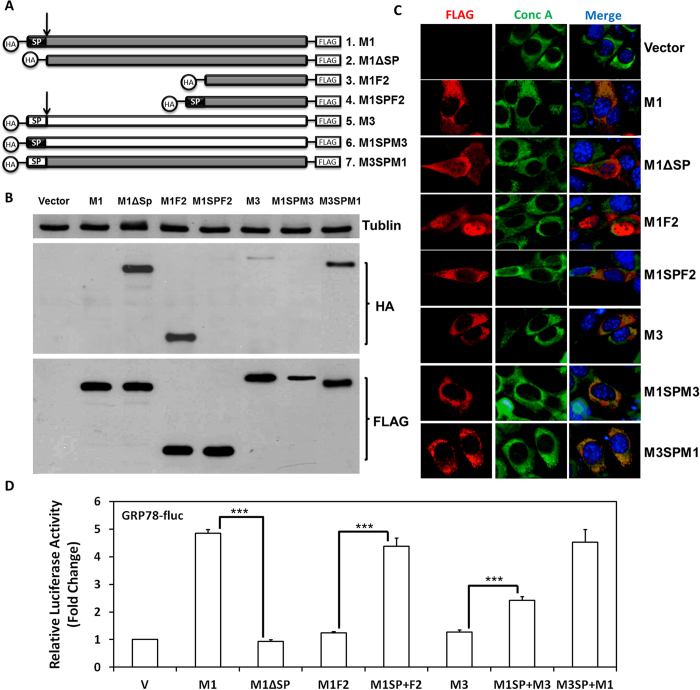
M1 requires ER localization for ER chaperone induction. (**A**) Schematic diagram of the M1 mutant constructs. **(B)** 293T cells were transfected for 24 hours with the plasmids encoding the indicated M1 mutants and harvested for western blot analysis using antibodies against tublin (top), HA (middle) or FLAG (bottom). **(C)** NIH3T3 cells transfected with the indicated M1 mutant constructs were fixed and analyzed by IFA using antibodies specific for FLAG (red), conc A (green) and Hoechst (Blue). **(D)** 293T cells were transfected with the GRP78-fluc, PGK-renilla-luciferase, and plasmids encoding each indicated M1 mutants. Cell lysates were collected 24 hours posttransfection and were analyzed by dual-luciferase assays as described in Fig. 1A.

We then asked whether the ER localization is required for M1 to induce chaperone genes. To test this hypothesis, we generated an M1 mutant (M1ΔSP) by removing the 18-aa signal peptide from the protein (Fig. 4A). M1 without the peptide lost its ER-specific distribution (Fig. 4C). Importantly, expression of M1ΔSP did not activate luciferase expression from the GRP78 promoter, indicating that the ER localization of M1 is required for its function (Fig. 4D). These results also rule out the possibility that M1 acts as a transcriptional activator of the chaperone genes, because even though M1ΔSP displayed an increased nuclear localization, it did not activate the chaperone gene.

To further assess the importance of the ER localization of M1, we constructed and analyzed the following mutants: a C-terminal fragment of M1 (M1F2); an M1F2 fused with the M1 signal peptide at its N-terminus (M1SPF2); an M3 mutant containing the M1 signal peptide (M1SPM3) and an M1 mutant containing the M3 signal peptide (M3SPM1) (Fig. 4A). All four clones were also engineered with an N-terminal HA tag and a C-terminal FLAG tag. This arrangement allowed us to determine whether the N-terminal cleavage took place due to the presence of the signal peptide. Cells were transfected with individual clones and subject to western blot analysis using HA and FLAG antibodies respectively (Fig. 4B). As expected, proteins bearing the M1 signal peptide sequence (i.e.: M1, M1SPF2, M1SPM3) could not be detected by the HA antibody (upper panel: Lane 1, 4, 6) indicating a cleavage at the N-terminus. In addition, IFA analysis revealed that all three proteins located to the ER (Fig. 4C). In contrast, clones without the signal peptide (M1ΔSP, M1F2) were detectable by both HA and FLAG antibodies (upper and lower panels: Lane 2, 3), and IFA analysis showed that these proteins are distributed throughout the cells with no specific ER localization (Fig. 4C). It is noteworthy that the HA antibody picked up a small amount of M3 protein suggesting that cleavage of M3 was incomplete when the protein is translated and processed (Lane 5). And the retarded cleavage became more evident in M3SPM1 expression (Lane 7) indicating that the M3 signal peptide causes slower cleavage than does that of M1.

Next, we performed reporter assays to determine how the mutations affected the function of M1/M3 on ER chaperone induction. 293T cells were transfected with each mutant construct and the GRP78-fluc reporter plasmid. Similar to what we observed with M1ΔSP, the M1F2 had no effect on the reporter activity. However, with the addition of M1 signal peptide, the mutant protein (M1SPF2) induced reporter activity to the same degree as wild type M1. More interestingly, while M3 had no effect on GRP78 expression (Fig. 1), by replacing its own signal peptide with that of M1, the M3 protein became a modest inducer of the GRP78 reporter (~a 2-fold increase). On the other hand, not surprisingly, even with a swapped signal peptide, the ER-localized M3SP+M1 was still capable of stimulating the GRP78 promoter (Fig. 4D).

Collectively, the above findings indicate that M1 acts through ER-to-nucleus signaling pathways and that the ER localization of M1 is indispensible for its function in ER chaperones induction. In addition, mutagenesis studies revealed that the M1 protein requires both its signal peptide and at least portion of its intraluminal fragment to reach the full potential of function.

### M1 activates the IRE1 and ATF6 axes of UPR to induce ER chaperone genes

ER chaperones are constantly expressed under all growth conditions. However a dramatic increase in their synthesis can be caused by activation of UPR in stressful conditions. Induction of different ER chaperone genes requires the cooperation between the three UPR signaling pathways^13^,^14^,^15^. Thereby, to specify the signaling branch(es) affected by M1, we set out to examine the status of each UPR signaling axis in M1-expressing cells.

**Figure 5:**
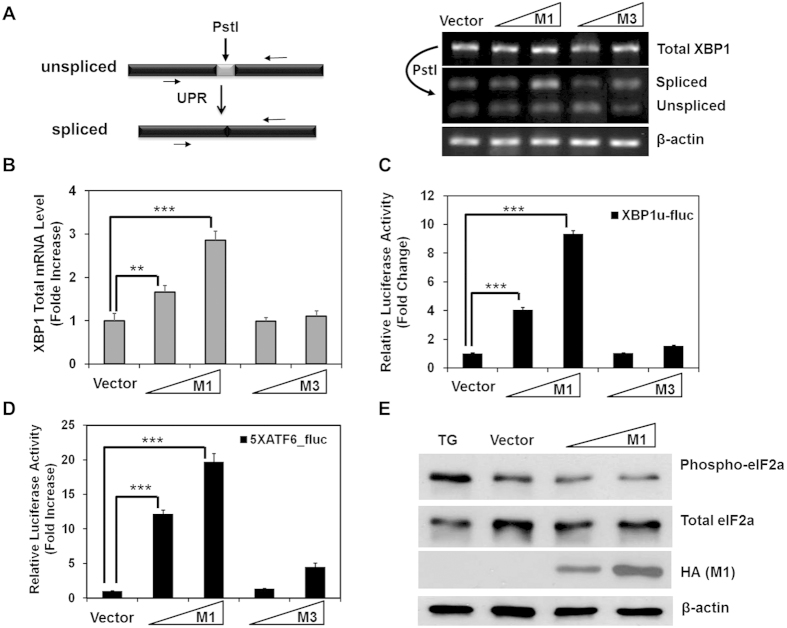
M1 activates the IRE1 and ATF6 pathways of UPR. (**A**) Left panel: analysis scheme for the splicing of XBP1 mRNA: the approximate location of the 26-nt intron, the PstI digestion site, and PCR amplification primers are shown. Right panel: the reverse transcripts of total XBP1 mRNA were analyzed by PstI digestion. Resulting DNA products were separated on 2% agrose gel. Transcripts of β-actin were included as loading controls. **(B)** The total XBP1 mRNA level was determined by quantitative RT-PCR. **(C)** Reporter assay was performed using the XBP1u-fluc (XBP-1 splicing reporter) and were analyzed as described in Fig. 1A. (**D**) Reporter assay was similarly performed using the 5XATF6-fluc plasmid. **(E)** 293T cells were transfected with plasmids encoding M1 or the control vector for 24 hours, treated with 20 nM Thapsigargin (TG) (lane 1) or DMSO (lane 2, 3, 4) for 30 minutes, and harvested for western blot analysis using antibodies specific for phosphorylated-eIF2a, total eIF2a and β-actin (loading control) as indicated.

To test whether M1 affects the IRE1 pathway, we probed for the unconventional splicing of XBP1 mRNA using RT-PCR and reporter assay. XBP1 cDNA was amplified using primers flanking the splicing sites and the PCR products were made subject to PstI digestion. PstI cuts a site within the 26-nt intron in the unspliced XBP1 (XBP1u) but leaves the spliced XBP1 (XBP1s) intact. In cells expressing M1, an elevated level of XBP1s was observed, manifested by an increased amount of spliced products that are resistant to PstI digestion (Fig. 5A). To quantitatively measure the splicing of XBP1, we employed a splicing-specific reporter system (pXBP1u-fluc). pXBP1u-fluc consists the coding sequence of firefly luciferase conjugated to the second ORF of XBP1u. Therefore, the luciferase is expressed only after IRE1-induced splicing removes the 26-nt intron^33^. Consistent with the PstI digestion results, M1 expression markedly increased the reporter activity to about 9-fold that of vector control (Fig. 5C).

When examining XBP1 splicing, we noticed that cells transfected with M1 expressed a higher level of total XBP1 mRNA than did vector or M3-transfected cells (Fig. 5B). Since XBP1 is produced downstream of activated ATF6, we suspected that the ATF6 pathway may also be stimulated by M1 expression. To determine if the ATF6 pathway is activated in response to M1, we used a reporter plasmid (p5XATF6-fluc) that contains five copies of ATF6 consensus binding site upstream of the firefly luciferase coding sequence^34^. M1 strongly upregulated the luciferase activity driven from the reporter (Fig. 5D), suggesting that ATF6 pathway was also activated by M1.

Finally, to investigate the PERK-mediated signaling in response to M1 expression, we examined the phosphorylation of eIF2α (p-eIF2α) induced by PERK upon UPR. To our surprise, p-eIF2α was not influenced by M1 expression (Fig. 5E). As a positive control, treatment with Thapsigargin (TG), an ER stress inducer, led to a significant increase in the ratio of p-eIF2α to total eIF2α, indicating the PERK-eIF2α pathway was functionally intact and our assay is valid.

### Infection by M1-deficient virus leads to reduced ER chaperone production

**Figure 6:**
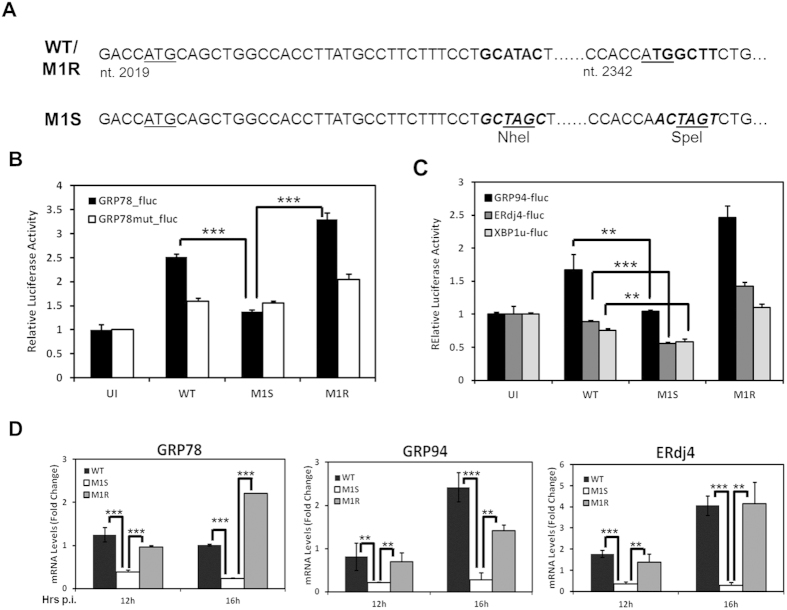
Infection by M1-deficient MHV-68 leads to reduced ER chaperon production. **(A)** Schematic diagram showing the construction of the M1stop MHV68 by introducing two stop codons (TAG) and two digestion sites (NheI, SpeI) into the M1 gene. **(B)** 293T cells were transfected for 24 hours with GRP78_fluc or GRP78mut_fluc and PGK_RL plasmids, and were mock infected or infected with wild-type (WT), M1stop (M1S) or M1 revertent (M1R) MHV-68 at MOI 5 for 18 hours. Cells were lysed and analyzed by dual-luciferase assay as in Fig. 1A. The ratio was calculated based on the unifected control. **(C)** Reporter assays were performed using the GRP94-fluc, ERdj4-fluc and XBP1u-fluc constructs. **(D)** NIH3T3 cells were mock infected or infected with WT, M1S or M1R MHV-68 at MOI 10. Cells were harvested at indicated time points for RNA extraction and analyzed by Quantitative RT-PCR using primer sets specific for indicated genes. The fold change is calculated based on the uninfected cells.

To extend the study on the importance of M1 in virus-mediate ER chaperone production, we constructed two recombinant MHV-68: An M1-stop virus (M1S) that contains two stop codons close to the N-terminal of the coding sequence (Fig. 6A) and a revertant virus of the M1S (M1R) in which the two stop codons were reverted back to wild type sequence to ensure what we observed with M1S viruses can be attributed to M1 deficiency rather than other unintentional mutations in the viral genome. Removal of the M1 expression from the MHV-68 genome had no effect on viral growth kinetics *in vitro* (Fig. S3) consistent with previous observations^24^,^28^.

293T cells transfected with GRP78-fluc reporter construct were either mock infected or infected with the wild type (WT), M1S or M1R MHV-68 at MOI 5. Cells were collected 18 hours post infection for dual-luciferase assays. Infection with WT and M1R virus led to a moderate increase in luciferase production, whereas M1S was not able to induce the reporter activity to an equivalent level (Fig. 6B). To further demonstrate that the observed phenotype is specific to the GRP78 promoter, cells were transfected with the GRP78mut-fluc plasmids and were identically infected. None of the viruses had a significant effect on the mutant reporter construct. We next performed similar tests with the other available reporter constructs including GRP94-fluc, ERdj4-fluc and XBP1u-fluc. In all cases, attenuated reporter activities were observed in cells infected with the M1S virus as compared to cells infected with WT and M1R MHV-68 (Fig. 6C).

To precisely define the kinetics of chaperone gene expression influenced by M1 during infection, the total RNA was harvested at indicated time points from NIH3T3 cells that were infected with WT, M1S or M1R viruses. The GRP78 mRNA level was determined by RT-PCR. Interestingly, prior to 8 hours post infection, the transcript levels of GRP78 remained comparable among all infected cells (Data not shown). However, at 12 and 16 hours post infection, times at which M1 protein becomes abundantly expressed (Figs 4, 5, 6A), the cells infected with M1S showed a major reduction of GRP78 transcript in comparison to cells infected with WT of M1R viruses (Fig. 6D).

It should be noted that infection by WT or M1R MHV-68 led to a lower induction of chaperone genes (Fig. 6B) in comparison to cells transfected with M1 gene coding plasmids (Figs 2A–4). One possible explanation is the different protein expression levels of M1 during infection versus transient over-expression; on the other hand, based on the screen results, we speculated that there are one or more viral factors that actually function to limit the signaling pathways involved in regulation of both the chaperone genes and other cellular factors unfavored in viral replication. It would be of interest to uncover how virus manipulates the chaperone network and its associated cellular machineries in order to achieve a balanced and beneficial outcome.

## Discussion

In present work, we identified M1 of MHV-68 that can efficiently induce the ER chaperone gene expression. In particular, we found that the ER-localized M1 functions through selective activation of the chaperone-inducing branches (IRE1 and ATF6) of UPR pathways while sparing the translation-inhibiting cascade (PERK).

**Figure 7:**
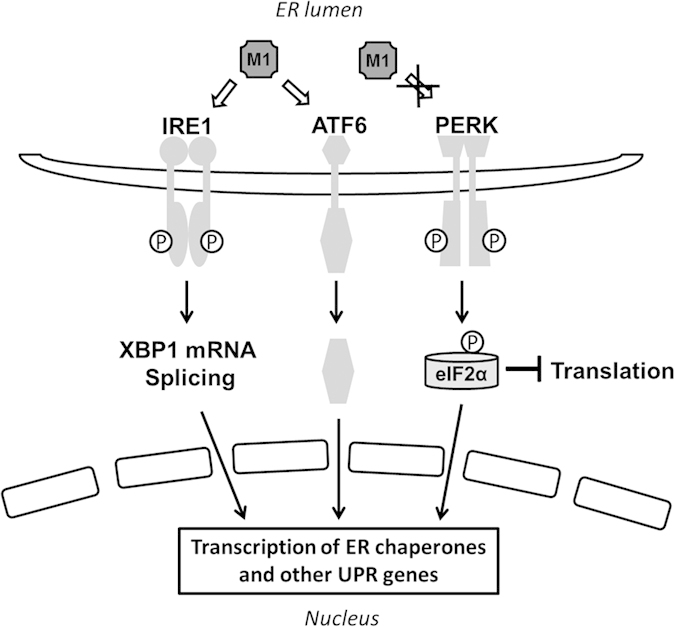
A scheme illustrating the effects of M1 on UPR signaling pathways. Our data demonstrate that the ER-localized M1 activates the IRE1 and ATF6 branches of UPR but spare the PERK axis to enhance the production of ER chaperone proteins.

M1 is a viral gene unique to MHV-68. Previous study has reported that disruption of the M1 gene led to enhanced reactivation of the virus in *in vivo* infection^24^. It was later reported by the same group that secreted M1 protein act as a viral superantigen and is responsible for Vβ4^+^ CD8^+^ T cell stimulation during MHV-68 infection in mice^28^. However, it has not been fully elucidated whether M1 plays a role inside the host cell. Here we discovered a novel function for the intracellular M1 protein. We found that M1 differentially modulate the UPR signaling cascades and preferentially induces the two chaperone-producing branches to activate ER chaperone expression. Specifically, the M1 protein stimulates the IRE1 and the ATF6 axes but spares the PERK pathway of UPR signaling (Fig. 7). It is of interest that the virus selectively acts on the beneficial aspects of the UPR program while avoiding the detrimental features. One possible explanation is that the virus takes advantage of different stress sensing or activation mechanisms deployed by the three ER transmembrane sensors (i.e. IRE1, ATF6 and PERK). Although it is still unclear how these signaling proteins sense the ER stress, a recent study used the three-dimensional structure analysis to demonstrate that IRE1 activation is actually caused by direct binding to the unfolded proteins rather than by chaperone association as suggested earlier on^9^. This finding implicates that the three branches of UPR signaling network may be distinctly modulated at the initiation stage. We propose that certain undefined properties in the M1 protein leads to the differentiated modulation on the UPR sensors. Therefore, using M1 as a tool may offer a unique opportunity to dissect the mechanism underlying UPR sensing and regulation.

It is worth mentioning that we also demonstrated that M1 requires ER localization for inducing chaperones, because the induction was lost by removal of the signal peptide, concomitantly with the loss of ER localization. More interestingly, the signal peptide of M1 enables non-inducing protein fragments to become activators of ER chaperone genes (Figs 3 and 4). We suspect that the 18-aa signal peptide of M1 protein plays an important role in switching on the IRE1 and ATF6 signaling axes through mechanisms that worth future investigation. Still, the presence of the intraluminal portion of M1 is critical to reach the full potential of its function in activating ER chaperone genes. Our hypothesis is that M1 requires the signal peptide to translocate to the action point, where the protein carries out its function.

It has been extensively reported that during a wide array of virus infections, cellular chaperones are elicited and play essential roles at various stages of the viral life cycle. Accumulating studies have shown that viruses are able to engage the host chaperone machinery to support effective cell entry and nuclear import^16^,^35^,^36^,^37^, viral genome replication^38^,^39^, viral protein expression and folding^17^,^18^,^19^, and virion assembly^40^,^41^,^42^. In addition, examples have been found wherein viruses pack the host chaperons into the virion core before egress and that the incorporated proteins are necessary for invading new cells^43^. More surprisingly, some viruses can even encode proteins that exhibit chaperone-like activities to facilitate their infection (e.g.: TAg of SV40)^44^. Furthermore, host chaperons are also utilized by the viruses to manipulate other cellular processes. Earlier studies have demonstrated that elevated levels of chaperone proteins can protect cells against apoptosis and confer resistance to cytotoxic and antimicrobial drugs^45^,^46^. Several reports further proposed that specific inhibition of the infection-induced chaperones may provide a solution to the appearance of drug resistant pathogens^47^. One recent study found that the inhibition of the Hsp90 chaperone *in vitro* and *in vivo* can prevent the outgrowth of EBV-transformed lymphoblastoid^48^. Moreover, a growing body of evidence suggests that improper activation of the UPR pathways and uncontrolled production of chaperones can adversely affect the immune response, a phenomenon exploited by certain viruses for an adaptive advantage^49^,^50^. To sum up, by augmenting the expression of chaperone proteins can create an environment favorable to virulence and may have become a survival tactic among different viruses. In response to such high demand of cellular chaperones during infection, viruses have the urge to evolve mechanisms to boost the chaperone production.

The fact that gamma-herpesviruses are capable of establishing lifetime persistence suggests that the viruses were evolved with ingenious skills to interact with their hosts. It is natural that different viruses adopt distinct approaches to tackle similar environmental challenges. For instance, previous studies have found that the M2 protein unique to MHV-68 enhances the cellular interleukin-10 (IL-10) expression to promote B cell growth and differentiation^51^ while EBV encodes a viral IL-10 homolog to achieve the same goal^52^. Another example is the regulation of cell cycle through cyclin D: MHV-68 and KSHV depend on encoding the conserved viral cyclins^53^ whereas EBV utilizes a viral gene (EBNA3C) to enhance the functional activity of cellular cyclin D1 for cell cycle progression^54^. Though relevant examples are lacking in regulation of UPR, previous work on human cytomegalovirus (beta-herpesvirus) has shown that viral infection can upregulate GRP78 expression without affecting the UPR pathways^55^, while studies on the herpes simplex virus type 2 (alpha-herpesvirus) revealed that the virus can encode a protein with chaperone-like activity. Therefore, although M1 is unique to MHV-68, we strongly believe that the human gamma-herpesviruses possess distinct chaperone-regulating approaches to achieve similar goals.

Our findings defined a new role for the M1 gene of MHV-68 in manipulating the host chaperone machinery. The ability of M1 to selectively activate the UPR signaling presents a unique opportunity for defining the sensing and activation mechanism of individual UPR pathway.

## Methods

### Cell lines

Human embryonic kidney 293T and Vero cells were maintained in Dulbecco’s modified Eagle medium (DMEM) supplemented with 10% fetal bovine serum, 100 U/ml of penicillin and 100 mg/ml of streptomycin (P/S). NIH3T3 cells were maintained in DMEM containing 10% bovine calf serum (BCS) and P/S.

### Plasmids

GRP78-fluc, GRP78mut-fluc and GRP94-fluc reporter constructs were kindly provided by Dr. Kazu Mori, Kyoto University, Kyoto, Japan^30^. The ERdj4-fluc plasmid was a gift from Dr. Laurie Glimcher, Harvard Medical School, Boston, MA^15^. The 5xATF6-fluc plasmid was provided by Dr. Ron Prywes, Columbia University, New York, NY^56^ and obtained via online purchase (Addgene plasmid 11976). The XBP1 splicing reporter plasmid (XBP1u-fluc) was provided by Dr. Yi-Ling Lin, Academia Sinica, Taipei, Taiwan, Republic of China^33^.

The wild-type M1 and M3 coding sequences (GenBank U97553) were PCR amplified from MHV-68 BAC DNA with an EcoRI site and Kozac sequence immediately upstream of the start codon and a c-terminal hemagglutinin (HA) tag before the stop codon and a BglII site downstream. The PCR fragments were cloned into a pCMV mammalian expression vector (clontech). Primer sequences used for M1 are 5′-GAATTCCACCATGCAGCTGGCCACCTTAT-3′ and 5′-GAAGATCTTTAAGCGTAATCTGGAA CATCGTATGGGTATCCTCCTCCTCCGGACTGCTGCCCAGG-3′. Primer sequences for M3 are 5′-GAATTCCACCATGGCCTTCCTATCCACA TCTG-3′ and 5′-GAAGATCTTTAAGCGTAA TCTGGAACATCGTATGGGTATCCTCCTCCTCCATGATCCCCAAAATACTCCAGC-3′. M1 and M3 mutants were similarly constructed using primer sequences listed in Table S1. Location prediction of signal peptides was performed on the SignalP 4.0 Server^57^ (http://www.cbs.dtu.dk/services/SignalP/).

### Reporter assays

293T Cells were grown to 60–75% confluency in 48-well plates and transfection was perforned using BioT (Bioland Scientific LLC) according to manufacturer’s instructions. Cells were lysed 24 hours posttransfection for dual-luciferase assays (Promega). For each assay, the firefly luciferase activity was normalized to the renilla luciferase reading in the same well, and the ratio was calculated based on the vector control (the value of which was set as 1).

### Construction of recombinant MHV-68

Recombinant MHV-68 was constructed using a two-step Red-mediated recombination method reported previously^58^. Briefly, the target sequence was divided into two fragments with an overlapping region of 100–200 bp. The two fragments were inserted upstream and downstream of a kanamycin-resistance cassette that contains an adjoining I-SceI site in a transfer plasmid with the backbone of pGEM-7zf(+). Using the resulting plasmid as a template, PCR was performed with primers bracketing the two sequence fragments and kanamycin-resistance cassette. The PCR product was subsequently digested with DpnI to eliminate the template plasmid, followed by gel extraction and electroporation into SHG68 competent cells harboring MHV-68 BAC, at 1.8 kV, 200 Ω, 25 μF (1 mm cuvette). Positive transformed clones were then made subject to second round Red recombination that removes the kanamycin-resistant cassette. The resulting Kan-sensitive clones were confirmed by sequencing and expanded for BAC DNA purification. The viral BAC DNA was transfected into 293T cells with an equal amount of plasmids expressing the Cre recombinase to remove the BAC sequence. Three days post transfection, single viral clones were isolated through limiting dilution, validated by PCR, and propagated. The primers used for constructing the recombinant virus are listed in Table S2.

### Electron microscopy

Cells were rinsed with PBS and fixed for 1 hr in 2% glutaraldehyde in PBS (pH 7.4) on ice. Cell pellets were collected and subjected to osmium post-fixation (1% OsO4 in PBS) for 1 hr (on ice), 2% uranyl acetate en bloc staining for 1 hr (on ice), followed by dehydration in an ascending ethanol series. The sample was infiltrated and embedded in Spurr’s resin and then sectioned about 75 nm using an UCT ultratome (LEICA). Sections were collected on naked grids (100 mesh, copper) and stained with saturated aqueous uranyl acetate and lead citrate from both sides. TEM imaging was performed using an FEI Tecnai TF20.

### Immunofluorescence assay

NIH3T3 cells were grown to 60%–75% confluency in 24-well plates and were either transfected with designated plasmids or infected with the M1-cHA MHV-68 at a multiplicity of infection (MOI) of 10. Transfected cells were fixed 24 hours posttransfection, and infected cells were fixed at indicated time points using 4% formaldehyde. Cells were then permeabilized and blocked in PBS containing 10% FBS, 0.5% BSA and 0.5% Triton-X for 1 hour at room temperature and incubated with primary antibodies overnight. Mouse anti-HA (Sigma) was used at 1:1000 dilution and the Alexa Fluor 488 conjugated anti-Concanavalin A (Invitrogen) at 1:100 dilution. Cells were washed and incubated with Alexa Fluor 594 goat-anti mouse IgG (Invitrogen) (1:1000) for 1 hour. The Hoechst dye was added for 5 minutes prior to analysis under a fluorescence microscope.

### Western blot analysis

Cells were harvested in lysis buffer (50 mM Tris pH 7.5, 150 mM NaCl and 1 mM EDTA, 1% NP-40 and 0.25% sodium deoxycholate) supplemented with protease inhibitors and phosphotase inhibitors where applicable. The protein lysates were then centrifuged, combined with 4 × protein sample buffer (0.25 M Tris pH 6.8, 40% glycerin, 20% β-mercaptoethanol, 8% SDS, 0.008% Bromophenol blue) and boiled for 5 minutes. The denatured proteins were separated by 10% SDS-PAGE and transferred to nitrocellulose membranes. Membranes were blocked for 1 hour in 5% non-fat, and incubated with one of the following primary antibodies: monoclonal mouse anti-KDEL (Assay Designs), monoclonal rabbit anti-phospho-eIF2a and mouse anti-total eIF2a (Cell Signaling), monoclonal mouse anti-HA and mouse anti-FLAG (Sigma), mouse polyclonal anti-ORF 26 and M9 (generated in our lab). The membranes were extensively washed and incubated with appropriate secondary antibodies conjugated to HRP (donkey anti-rabbit or anti-mouse IgG, GE Healthcare) and developed by the Western Lighting system (Perkin-Elmer).

### PCR (regular and real-time)

Total RNA from cultured cells was insolated using RNA Mini Kit (Invitrogen) and reverse transcribed into cDNA using qScript cDNA synthesis Kit (Quantas). Real-time PCR was performed using the specific primer sets (Table 1). For XBP-1 splicing assays, the XBP-1 cDNA was PCR-amplified using the indicated primers and were then made subject to PstI digestion. Digestion of the unspliced XBP1 produces two DNA fragments (291 bp and 307 bp). The final DNA products were resolved on a 2.5% agrose gel, stained with ethidium bromide and visualized by UV.

## Additional Information

**How to cite this article**: Feng, J. *et al.* M1 of Murine Gamma-Herpesvirus 68 Induces Endoplasmic Reticulum Chaperone Production. *Sci. Rep.* **5**, 17228; doi: 10.1038/srep17228 (2015).

## Supplementary Material

Supplementary Table S1, Table S2, Figure S1, Figure S2, Figure S3

## Figures and Tables

**Table 1:** Primer sequences for quantitative RT-PCR.

Taget Gene	Direction	Primer Sequence (5′ to 3′)
GRP78	Forward	CGGGCAAAGATGTCAGGAAAAG
	Reverse	TTCTGGACGGGCTTCATAGTAGAC
GRP94	Forward	GAATGCTTCGCCTCAGTTTG
	Reverse	TCATCTTCGTCTTGCTCTGTG
ERdj4	Forward	AAAATAAGAGCCCGGATGCT
	Reverse	CGCTTCTTGGATCCAGTGTT
XBP1	Forward	CTGGAACAGCAAGTGGTAGA
	Reverse	CTGGATCCTTCTGGGTAGAC
GAPDH	Forward	TGCACCACCAACTGCTTAGC
	Reverse	GGCATGGACTGTGGTCATGAG
Actin	Forward	CACCCACACTGTGCCCATCTAC
	Reverse	GTGAGGATCTTCATGAGGTAGTC
