# Supplementary material for: M1 of Murine Gamma-Herpesvirus 68 Induces Endoplasmic Reticulum Chaperone Production
Source: Sci Rep. 2015 Nov 30;5:17228. doi: 10.1038/srep17228 (PMC4663489; doi:10.1038/srep17228)
Supplement: Supplementary Table S1, Table S2, Figure S1, Figure S2, Figure S3 [file srep17228-s1.doc]

­­*Supplemental materials*

**M1 of Murine Gamma-herpesvirus 68 Induces Endoplasmic Reticulum Chaperone Production**

Jiaying Feng, Danyang Gong, Xudong Fu, Ting-ting Wu, Jane Wang, Jennifer Chang, Jingting Zhou, Gang Lu, Yibin Wang, Ren Sun

**Experimental Procedures**

*Plaque assay –* 10-fold serial dilutions of the concentrated viruses were inoculated on Vero cells for 1 hour. The infected cells were then overlaid with 5% methylcellulose DMEM containing 10% FBS and P/S for 6 days. Cells were fixed using 2% crystal violet in 20% ethanol. Plaques were counted at the optimal dilutions to calculate virus titer.

**Table S1: Primer sequences for constructing M1/M3 mutant clones**

| **Name** | **Direction** | **Primer Sequence (5’ to 3’)** |
| --- | --- | --- |
| **M1** | Forward | GAATTCAAATGCAGCTGGCCACCTTAT |
| **M1ΔSP** | Forward | GAATTCAAATGTCCTGCATACTGGGCCAAAG |
| **M1_F2** | Forward | GAA TTC AAATGGACCCTGACTGCCAGT |
| **M1SP+F2** | Forward | GAATTCAAATGCAGCTGGCCACCTTATGCCTTCTTTCCTGCATACTGGG  -CCAAAGCATAGCTCACTGGGACCCTGATCTGCCAGT |
| **M3** | Forward | GAATTCAAATGGCCTTCCTATCCACATCTG |
| **M1SP+M3** | Forward | GAATTCAAATGCAGCTGGCCACCTTATGCCTTCTTTCCTGCATACTGGG  -CCAAAGCATAGCTCACTGGCTAGGTTTGGCACCTGCTCT |
| **M3SP+M1_F1** | Forward | GAATTCAAATGGCCTTCCTATCCACATCTGTGCTCATTAAATGCTGCATC  -CTCCTGTT |
| **M3SP+M1_F2** | Forward | AAATGCTGCATCCTCCTGTTGGCAGGAGGATTGGCTGAGAGCCACTGGC  -CATCTGTGGTC |
|  |  |  |
| **M1_FLAG** | Reverse | GAAGATCTTTACTTGTCATCGTCATCCTTGTAATCGGACTGCTGCCCAGG |

Table S2: Primer sequences for constructing recombinant MHV-68

| **Name** | **Direction** | **Primer Sequence (5’ to 3’)** |
| --- | --- | --- |
| **EcoRI_M1cHA_LF** | Forward | GGAATTCAAATTATGGCTTCTTTAGAACCCGAC |
| **M1cHA_LF_R** | Reverse | TTAAGCGTAGTCTGGGACGTCGTATGGGTATCCTCCTCCTCCGGACTGCTGCCCAGG |
|  | Forward | GGAGGAGGAGGATACCCATACGACGTCCCAGACTACGCTTAAGCCTGAATACATGTTTACTGGGG |
| **BgIII_M1cHA_LF** | Reverse | GGAAGATCTTTAATGGACTGAAGCCCGTCA |
| **SalI_M1cHA_RF** | Forward | ACGCGTCGACGAACAAAGTTCTTTTACTGCAGAAAGC |
| **BamHI_M1cHA_RF** | Reverse | CGCGGATCCGCTTACAACCTACGCGGC |
| **EcoRI_M1s_LF** | Forward | GGAATTCAGATGCGTAAGGAGAAAATACCGC |
| **M1S_LF1_NheI** | Reverse | GCTTTGGCCCAGCTAGCAGGAAAGAAGGC |
| **M1S_LF1_NheI** | Forward | GCCTTCTTTCCTGCTAGCTGGGCCAAAGC |
| **M1S_LF2_SpeI** | Reverse | GAGGTGGCAGACTAGTTGGTGGGTAG |
| **M1S_LF2_SpeI** | Forward | CTACCCACCAACTAGTCTGCCACCTC |
| **BgIII_M1s_LF** | Reverse | GGAAGATCTATCCAGCAAAGGTGAGATCCAG |
| **SalI_M1s_RF** | Forward | ACGCGTCGACCCACCTCAGGAAGGGTCA |
| **BamHI_M1s_RF** | Reverse | CGCGGATCCTGGTGGCACCCATCTTGATG |
|  |  |  |

**Figure S1.**

**
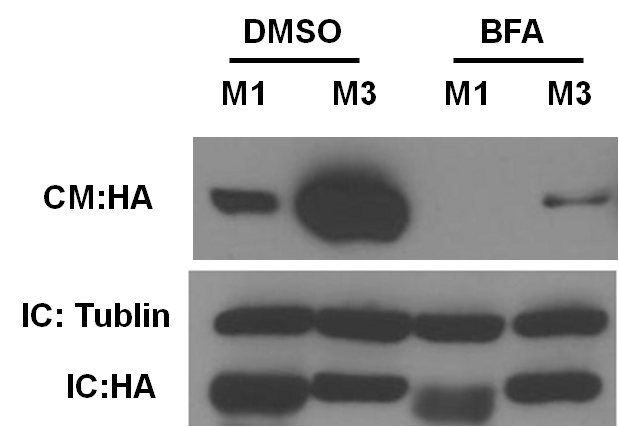
**

**Figure S1: M1 and M3 are secreted protein.** 293T cells were transiently transfected with the M1 or M3 expression plasmids and were treated with 10ug/ml of brefeldin A (BFA) (Sigma) or DMSO as solvent control. BFA is a strong inhibitor of protein secretion. 24 hours post treatment, proteins were collected from the culture medium (CM) or cells (IC: intracellular) respectively, and were analyzed by western blot using anti-HA antibody to probe for M1 and M3 proteins and anti-tublin for an internal control.

**Figure S2.**

**
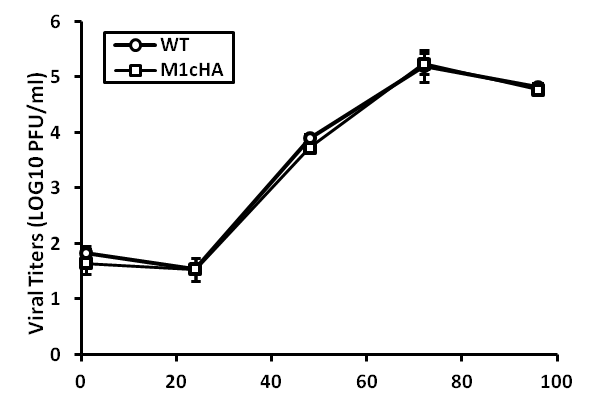
**

**Figure S1: The multi-step growth curve of M1cHA MHV-68.** NIH3T3 cells were infected with wild-type (WT) and M1cHA MHV-68 at MOI 0.05 and harvested at 1, 24, 48, 72 and 96 hours post infection. Following three freeze-thaw cycles of the entire cell and supernatant lysate, viral titers were determined by plaque assay.

**Figure S3.**

**A**

**
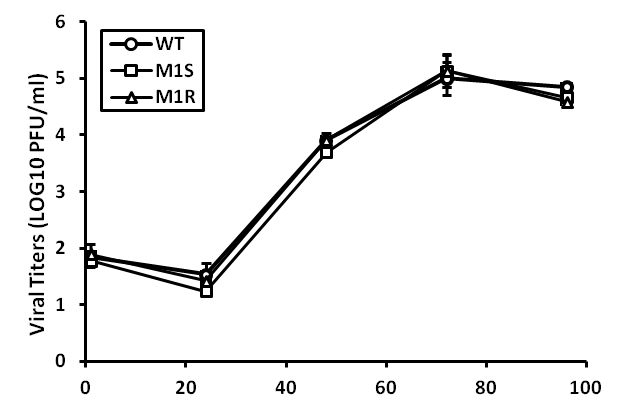
**

**B**


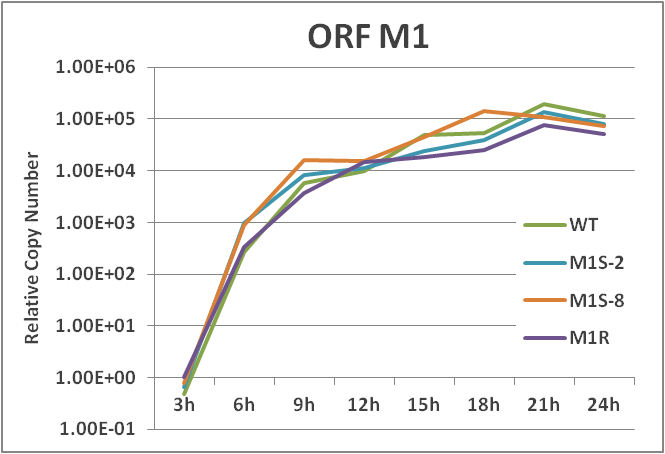

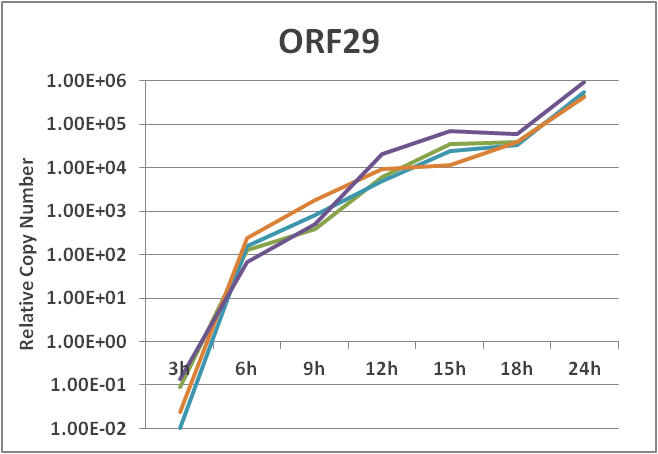

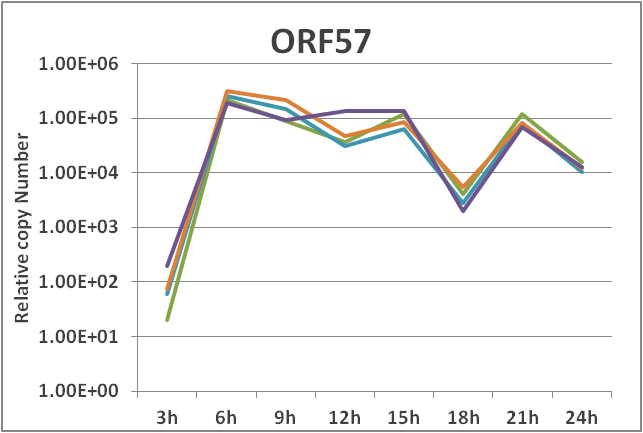

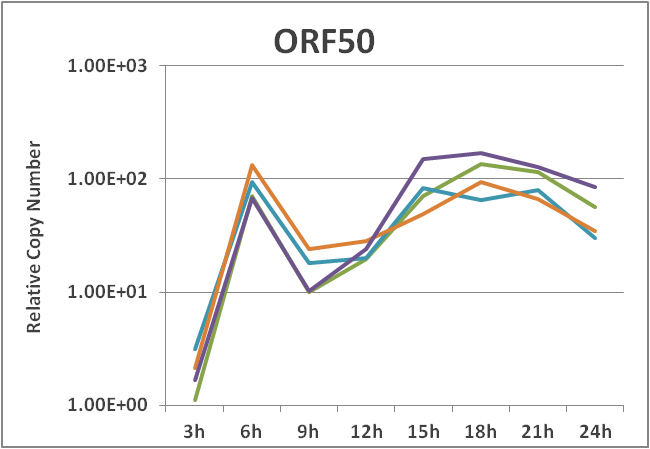


**Figure 4-10: Multi-growth curve and viral gene transcription of WT, M1-stop (M1S) and M1-revertant (M1R) MHV-68.** NIH3T3 cells were infected with indicated MHV-68 at MOI 0.05 and harvested at 1, 24, 48, 72 and 96 hours post infection. Following three freeze-thaw cycles of the entire cell and supernatant lysate, viral titers were determined by plaque assay. (B) Comparison of the immediate early, early and late gene expression among WT, M1-stop (M1S) and M1-revertant (M1R) MHV-68 using RT-Q-PCR
